# Population Pharmacokinetic Modeling of Intravenous Fluconazole to Inform eGFR-Based Dosing: A Simulation Study Demonstrating Superiority over Standard Regimens

**DOI:** 10.3390/pharmaceutics18080929

**Published:** 2026-07-29

**Authors:** Terezie Macková, Martin Vodička, Ondřej Slanař, Martin Šíma

**Affiliations:** 1 Department of Clinical Pharmacy, Tomas Bata Hospital Zlin, 762 75 Zlin, Czech Republic; martin.vodicka@bnzlin.cz; 2 Department of Pharmacology, First Faculty of Medicine, Charles University and General University Hospital in Prague, 128 00 Prague, Czech Republic; ondrej.slanar@lf1.cuni.cz (O.S.); martin.sima@lf1.cuni.cz (M.Š.)

**Keywords:** fluconazole, glomerular filtration rate, Monte Carlo simulation, nonlinear mixed-effects modeling, PK/PD target, therapeutic drug monitoring

## Abstract

**Aim:** This study aimed to develop a population pharmacokinetic (PK) model of intravenous fluconazole and, using model-based simulations, propose initial dosing recommendations that optimize efficacy while limiting toxicity. **Methods:** Therapeutic drug monitoring data from 116 adult patients (183 serum concentrations) treated with intravenous fluconazole between 2022 and 2025 at a single center were analyzed using nonlinear mixed-effects modeling. Monte Carlo simulations were applied to identify dosing strategies that maximize the probability of achieving the PK/PD efficacy target. **Results:** Estimated glomerular filtration rate (eGFR) was the strongest predictor of fluconazole clearance, and adjusted body weight (ABW) influenced the volume of distribution. For a patient with ABW of 75 kg and eGFR of 90 mL/min, the estimated volume of distribution and clearance were 41.6 L and 0.81 L/h, respectively. An eGFR-based dosing nomogram was developed, and simulations demonstrated that this approach outperformed standard dosing regimens in achieving the PK/PD target. **Conclusions:** These findings suggest an initial loading dose (2.8 times the maintenance dose) followed by individualized, eGFR-guided maintenance dosing to enhance the safety and efficacy of intravenous fluconazole therapy; however, prospective clinical validation is required before routine clinical implementation.

## 1. Introduction

Invasive candidiasis has been a significant clinical challenge in critically ill patients, with crude mortality rates of 40–60% despite receiving antifungal therapy and attributable mortality up to 40% [1,2]. Initiating appropriate antifungal therapy within 24–48 h can lead to a reduction in mortality by up to 50%, highlighting the importance of adequate dosing [3]. Fluconazole, a triazole antifungal, plays a key role in both the treatment and step-down therapy of invasive candidiasis, particularly for fluconazole-susceptible species or echinocandin-resistant organisms [2,4]. Fluconazole is a weakly basic, hydrophilic compound with low plasma protein binding (~11–12%), excellent oral bioavailability (~90%) after oral administration, and predictable intravenous kinetics, with a volume of distribution of approximately 0.7 L/kg, reflecting distribution throughout total body water [5,6,7]. Fluconazole has a long half-life of around 30 h in healthy patients, and approximately 80% is eliminated renally as an unchanged drug [6]. Its pharmacodynamic efficacy is best predicted by the ratio of the 24 h area under the curve (AUC_24_) to the minimal inhibition concentration (MIC), which has been used as a pharmacokinetic/pharmacodynamic (PK/PD) target in a recent study [8,9,10,11].

Although therapeutic drug monitoring (TDM) is well established for several mold-active azoles, current clinical guidelines neither recommend nor discourage its routine use for fluconazole because of its generally predictable pharmacokinetics and wide therapeutic index [8,10]. Nevertheless, this places greater emphasis on selecting an appropriate initial dosing regimen, particularly in critically ill patients, in whom pharmacokinetic variability may compromise target attainment. Therefore, optimization of the initial dose using patient-specific characteristics represents an attractive strategy to improve target attainment before TDM is considered.

Despite established pharmacodynamic targets, adequate drug exposure is frequently not achieved in critically ill patients, as shown by several recent studies [1,4,12,13]. These findings demonstrate that standard dosing regimens often lead to substantial underexposure in this population. Current dosing guidelines rely on simplified dose reductions based on estimated glomerular filtration rate (eGFR), which do not fully capture this relationship or provide quantitative guidance across the spectrum of renal function [14,15].

Population pharmacokinetic modeling and Monte Carlo simulations offer a rational approach to address this challenge. Sandaradura et al. showed that model-optimized dose selection enabled ≥98% of patients to achieve efficacy targets throughout treatment while minimizing overexposure, supporting dose individualization from therapy initiation without requiring TDM [13]. Similarly, Van Daele et al. reported that model-informed dosing strategy resulted in ≥98% probability of target attainment (PTA) [16].

The objective of this study was to develop a robust population pharmacokinetic model of intravenous fluconazole and, using model-based predictive simulations, to propose initial dosing recommendations that balance efficacy and safety by optimizing the probability of achieving the PK/PD efficacy target while constraining drug exposure within the toxicity threshold.

## 2. Materials and Methods

### 2.1. Study Design and Ethics

This retrospective, single-center, observational study included adult patients (≥18 years) hospitalized at Tomas Bata Hospital Zlin, Czech Republic between 1 January 2022 and 31 March 2025 who were treated with intravenous fluconazole and had at least one measured fluconazole serum concentration during the study period. Patients were excluded if they were undergoing renal replacement therapy or if relevant clinical data were missing from medical records.

The study was approved by the Ethics Committee of Tomas Bata Hospital Zlin (Ref. No. 2025-23) on 12 August 2025, and was conducted in accordance with the principles of the Declaration of Helsinki. Upon hospital admission, all patients provided general informed consent, which included consent for the use of anonymized clinical data for research purposes.

Eligible patients were identified retrospectively from hospital medical records, from which demographic, clinical, laboratory, treatment, and TDM data were subsequently extracted as described below.

### 2.2. Data Retrieval

The following data were collected form the medical records: demographic characteristics [age, sex, actual body weight and height during the fluconazole administration period, body surface area (BSA), adjusted body weight (ABW) and hospital ward], serum fluconazole concentrations measured by the biochemistry laboratory, details of fluconazole therapy (treatment duration, administered doses, route of administration, dosing regimen, timing of administration, infusion duration, and timing of TDM), markers of drug elimination [serum creatinine, creatinine-based estimation of glomerular filtration rate (eGFR), serum cystatin C, cystatin-based eGFR], indication for fluconazole treatment, and the use of renal replacement therapy.

Creatinine-based eGFR was calculated using the CKD-EPI 2021 creatinine equation, while cystatin C-based eGFR was calculated using the CKD-EPI 2012 cystatin C equation [17]. ABW was calculated as

ABW = IBW + 0.4 × (TBW − IBW)

where TBW is total body weight. IBW (ideal body weight) was calculated according to the Devine formula (50 kg + 2.3 kg for each inch over 5 feet for men and 45.5 kg + 2.3 kg for each inch over 5 feet for women).

### 2.3. Population Pharmacokinetic Analysis

Nonlinear mixed-effects analyses of fluconazole serum concentration–time profiles were conducted with the Monolix Suite (version 2021R2; Lixoft SAS, Antony, France), which employs the Stochastic Approximation Expectation Maximization algorithm. Candidate structural models included one- and two-compartment formulations incorporating either first-or zero-order elimination. All model parameters were assumed to follow log-normal distributions. Inter-individual variability was evaluated for each pharmacokinetic parameter using log-normally distributed random effects with estimable variance. Residual unexplained variability was explored using constant, proportional, or combined error models. The optimal structural model was chosen according to predefined criteria: reductions in the objective function value (OFV), differences in the Akaike and Bayesian information criteria (AIC, BIC), visual appraisal of goodness-of-fit (GOF) diagnostics, and acceptable relative standard errors (R.S.E.) of parameter estimates.

In a subsequent step, potential covariate effects on the base model were explored. Age, height, total body weight, ABW, and BSA were examined as continuous covariates, whereas sex was evaluated as a categorical covariate. Time-varying covariates—serum creatinine, serum cystatin C, and both creatinine-based and cystatin C-based eGFR—were tested by incorporating them as regressors. Initial covariate screening was performed using graphical exploration and univariate associations between covariates and individual pharmacokinetic parameter estimates, assessed through Pearson’s correlation. Covariates identified as potentially relevant were subsequently verified or dismissed via manual stepwise forward inclusion and backward elimination. A decrease in OFV of >3.84 points between nested models (χ^2^ test, α = 0.05, 1 degree of freedom) signified statistical significance for inclusion. During backward elimination, covariates were retained if removing them increased the OFV by more than 6.64 points (*p* < 0.01). Additional considerations in final model selection included reasonable R.S.E. values, biological plausibility of parameter–covariate relationships, and lack of systematic trends in GOF plots.

The final model was evaluated using individual model fitting curves, GOF diagnostics (observed concentrations vs. individual and population predictions), and internal validation via a visual predictive check (VPC). The VPC compared the observed data with the 10th, 50th, and 90th percentiles of simulated concentration–time profiles generated from 1000 replicates. Model robustness was further examined using a bootstrap procedure in which 500 resampled datasets were created. For each bootstrap replicate, model parameters were re-estimated using the Rsmlx package for Monolix Suite (version 2021R2; Lixoft SAS, Antony, France). The median and 95% confidence intervals of bootstrap-derived parameter estimates were then contrasted with those obtained from the final model.

### 2.4. Monte Carlo Simulations

The final population pharmacokinetic model for fluconazole was imported into Simulx (version 2021R2;Lixoft SAS, Antony, France) to perform Monte Carlo simulations (500 replicates of the original dataset). These simulations were used to characterize the theoretical population distribution of concentration–time profiles under several dosing strategies: the standard approved regimens for cryptococcosis (a 400 mg loading dose followed by 200 mg once daily, each administered as a 1 h intravenous infusion) and for invasive candidiasis (an 800 mg loading dose followed by 400 mg once daily, via 1 h intravenous infusion), as well as an off-label high-dose regimen (400 mg every 12 h, infused over 1 h).

An AUC_24_/MIC ratio ≥ 100 was adopted as the PK/PD target for efficacy [8], whereas an AUC_24_ exceeding 1500 mg·h/L was considered the toxicity threshold [18]. The probabilities of target attainment (PTA) for both efficacy and toxicity were estimated for all dosing regimens using MIC values of 2 mg/L and 4 mg/L, corresponding to the EUCAST non-species-related breakpoints for susceptibility and resistance, respectively [8,19].

Subsequently, administration of eGFR-adjusted fluconazole dosing [based on the identified effect of eGFR on fluconazole clearance (CL)] was simulated to maximize the probability of attaining the efficacy target while minimizing the probability of exceeding the predefined toxicity threshold. For this purpose, the relationship between eGFR and fluconazole CL, described in the final model, was translated into a dosing nomogram for the determination of the optimal daily fluconazole dose according to the patient’s eGFR. The nomogram was designed to achieve an AUC_24_ of 850 mg·h/L, corresponding to the midpoint of the therapeutic exposure range bounded by the efficacy target (AUC_24_/MIC ≥ 100 for MIC = 2 mg/L; AUC_24_ = 200 mg·h/L) and the predefined toxicity threshold (AUC_24_ = 1500 mg·h/L).

### 2.5. Individual Data Processing and Statistics

Descriptive parameters—medians, interquartile ranges (IQRs) and 95% confidence intervals (CI) for medians—were calculated using GraphPad Prism 8.2.1 software (GraphPad Inc., La Jolla, CA, USA). Individual values of the AUC_24_ and trough levels (C_min_) on the 10th day of fluconazole therapy (expected steady state) at an approved dosing of 200 mg once daily via 1 h intravenous infusion were estimated as the Empirical Bayes Estimates in Simulx software 2021R2 and then the relationship between AUC_24_ and C_min_ was evaluated using the linear regression model in GraphPad Prism 8.2.1 software.

## 3. Results

### 3.1. Study Population

A total of 116 patients [38 (32.8%) females, 78 (67.2%) males] were eligible for inclusion in pharmacokinetic analysis. The most frequent diagnoses for fluconazole treatment were Candida infections based on microbiological cultivations, namely *Candida albicans* (*n* = 75; 72.4%), *Candida glabrata* (*n* = 5; 4.3%), *Candida tropicalis* (*n* = 5; 4.3%), *Candida parapsilosis* (*n* = 2; 1.7%), *Candida albicans + Candida glabrata* (*n* = 3; 2.6%), and *Candida albicans + Candida tropicalis* (*n* = 3; 2.6%). In 14 patients (12.1%) fluconazole was used as part of empirical antimicrobial therapy.

The majority of patients (*n* = 96; 82.8%) were hospitalized in various intensive care units (ICUs), namely the Department of Anesthesiology, Resuscitation and Intensive Medicine (*n* = 78; 67.2%), the Internal Medicine ICU (*n* = 6; 5.2%), the Oncology ICU (*n* = 5; 4.3%), the Cardiology ICU (*n* = 4; 3.4%), the Pulmonary ICU (*n* = 2; 1.7%), and the Neurology ICU (*n* = 1; 0.9%). The remaining 20 patients (17.2%) were hospitalized in standard wards, including the Internal Medicine ward (*n* = 6; 5.2%), the Surgical ward (*n* = 4; 3.4%), the Pulmonary ward (*n* = 3; 2.6%), the Oncology ward (*n* = 2; 1.7%), the Neurosurgery ward (*n* = 1; 0.9%), the Ophthalmology ward (*n* = 1; 0.9%), the Rehabilitation ward (*n* = 1; 0.9%), and the Oral, Maxillofacial and Facial Surgery ward (*n* = 1; 0.9%).

Demographic, laboratory and clinical data of patients are summarized in Table 1. Clinically relevant augmented renal clearance was uncommon in the study cohort; only four patients (3.4%) had creatinine-based eGFR values ≥130 mL/min/1.73 m^2^.

Fluconazole was administered as a part of routine clinical practice at a median (min–max) dose of 400 (200–800) mg, every 12 or 24 h via 30 min, 60 min or 120 min intravenous infusion.

A total of 183 measured serum fluconazole concentrations (1–4 per patient; average 1.6 per patient) were included in the analysis. Thirty samples (16.4%) were collected as trough levels (0–2 h before the next dose), 9 samples (4.9%) were collected as peak levels (0–2 h after the start of infusion), and 144 samples (78.7%) were obtained during the dosing interval, with the exact sampling time recorded.

### 3.2. Population Pharmacokinetic Analysis

A one-compartment model with linear elimination and a proportional residual error model was identified as the best structural and error model to describe the fluconazole concentration–time data and account for residual variability. The pharmacokinetic model was parameterized using volume of distribution (Vd) and CL.

Preliminary graphical exploration indicated that, in the subgroup of patients for whom cystatin C was available (*n* = 53), the predictive performance of creatinine-based and cystatin C-based eGFR estimates was essentially equivalent (Supplementary Figure S1). Therefore, creatinine-based eGFR was selected, allowing the population model to be developed using the full cohort (*n* = 116).

Covariate analysis showed that eGFR was the most appropriate covariate for fluconazole CL, while ABW best described variability in Vd. Their relevance was supported by significant decreases in OFV (32.34 and 21.15 points, respectively) and by physiological plausibility. After incorporating these covariates into the model, none of the remaining tested variables demonstrated a meaningful influence on fluconazole pharmacokinetics.

The final equations describing the relationships between fluconazole pharmacokinetic parameters and their covariates, together with the population parameter estimates and bootstrap results, are summarized in Table 2. In the final model, an individual with normal renal function (eGFR = 90 mL/min) and a median ABW of 75 kg had a predicted Vd of 41.58 L and a CL of 0.81 L/h, corresponding to an elimination half-life of 35.6 h.

Diagnostic GOF plots (Supplementary Figure S2) demonstrated an adequate fit of the final covariate model to the observed fluconazole concentrations. The VPC plot (Supplementary Figure S3) showed that the final model appropriately captured both the central tendency and variability of the observed concentration–time profiles. As presented in Table 2, the R.S.E. (maximum 30.3%) indicated that all pharmacokinetic parameters were estimated with acceptable precision. Median parameter estimates obtained from 500 bootstrap replicates were in close agreement with the final model values (maximum deviation 16%), further supporting the robustness of the parameter estimates. The η-shrinkage values for the estimated random effects were negligible (CL 0.26%, Vd −0.55%, β_CL_eGFR 0.27%), indicating reliable individual Empirical Bayes Estimates and supporting the validity of the diagnostic plots and covariate analysis.

**Figure 1:**
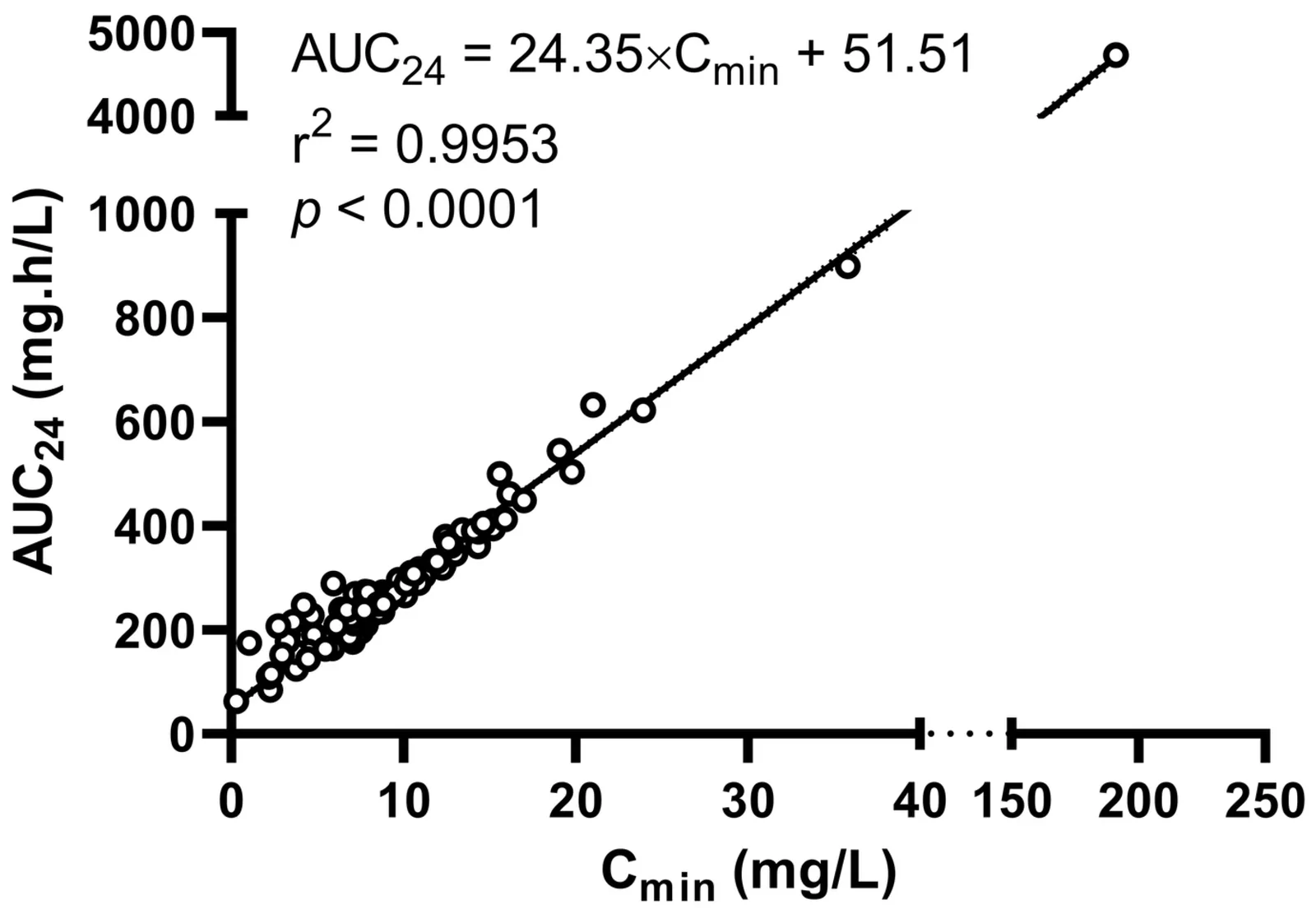
Relationship between fluconazole trough (C_min_) concentrations and exposures (AUC_24_) at steady state (10th day of therapy) based on individual fluconazole pharmacokinetic profiles after simulated administration of 200 mg every 24 h via 1 h infusion using the Empirical Bayes Estimates.

Simulation based on the Empirical Bayes Estimates of individual fluconazole pharmacokinetic profiles showed an excellent association of steady-state fluconazole trough concentrations (C_min_) and fluconazole exposure (AUC_24_) (Figure 1).

### 3.3. Monte Carlo Simulation

**Figure 2:**
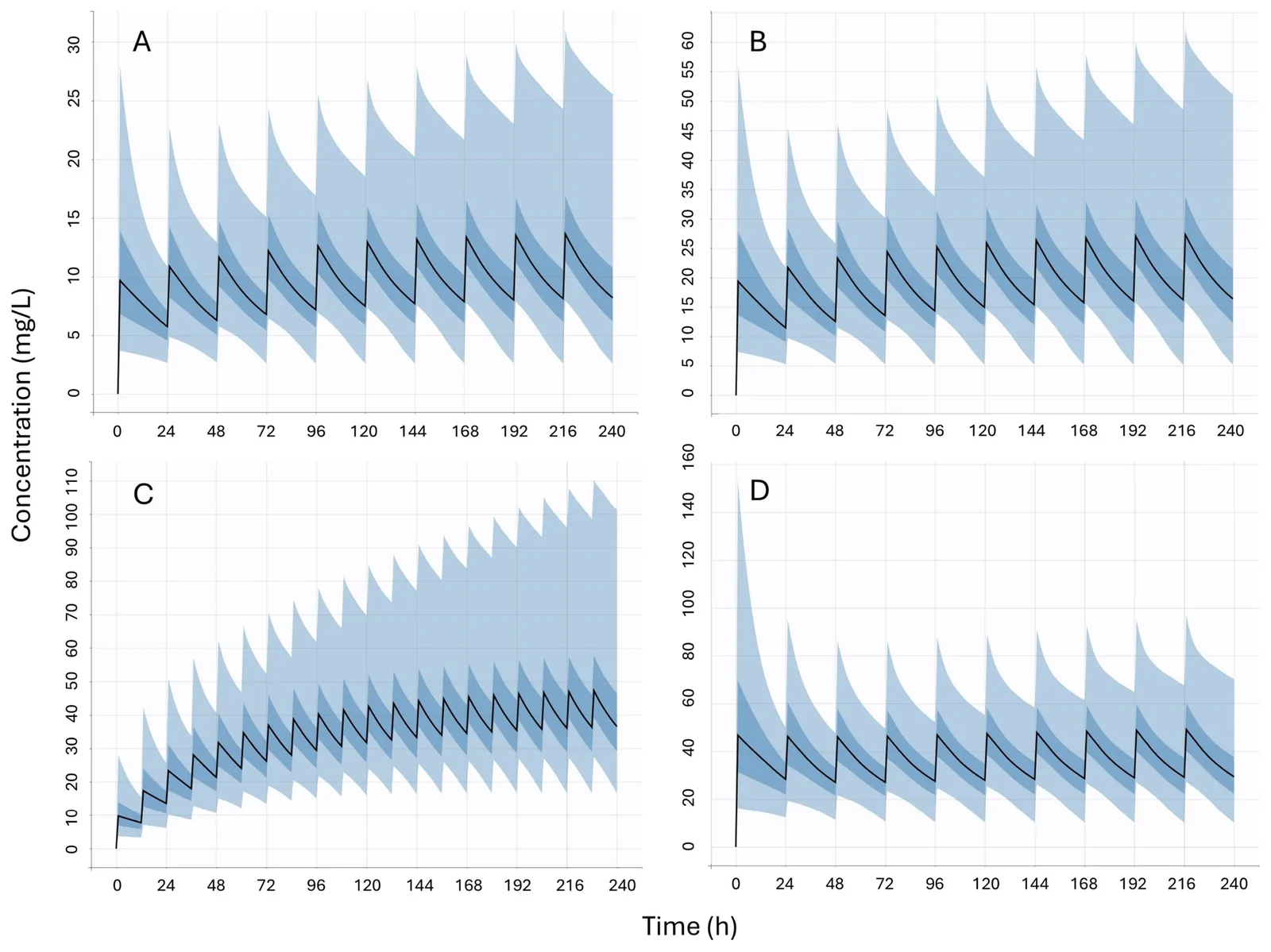
The Monte Carlo simulation of the fluconazole serum concentration–time profiles following fluconazole intravenous administration: (**A**) loading dose of 400 mg, followed by maintenance dose of 200 mg once daily via 1 h intravenous infusion; (**B**) loading dose of 800 mg, followed by maintenance dose of 400 mg once daily via 1 h intravenous infusion; (**C**) 400 mg every 12 h via 1 h intravenous infusion; (**D**) eGFR-based maintenance dose (according to proposed nomogram) once daily via 1 h intravenous infusion with an initial loading dose of 2.8-fold higher than the maintenance dose. The black line represents the median, and the four blue bands represent the percentiles (5–27.5%, 27.5–50%, 50–72.5% and 72.5–95%) of the 90% simulated concentration distribution.

Figure 2 illustrates the simulated theoretical distributions of fluconazole serum concentration–time profiles for four alternative dosing strategies. First, simulations were performed for the standard approved regimens used in cryptococcosis (Figure 2A) and invasive candidiasis (Figure 2B), and further for an off-label high-dose regimen (Figure 2C). To subsequently maximize the PTA for efficacy while minimizing the PTA for toxicity, a dosing approach individualized according to the patient’s eGFR—identified as a covariate of fluconazole CL—was evaluated (Figure 2D). Based on the final population pharmacokinetic model, the required maintenance dose (MD) was described by the following equation:

MD (mg/day) = 769.5 × (eGFR/90)^0.43^

**Figure 3:**
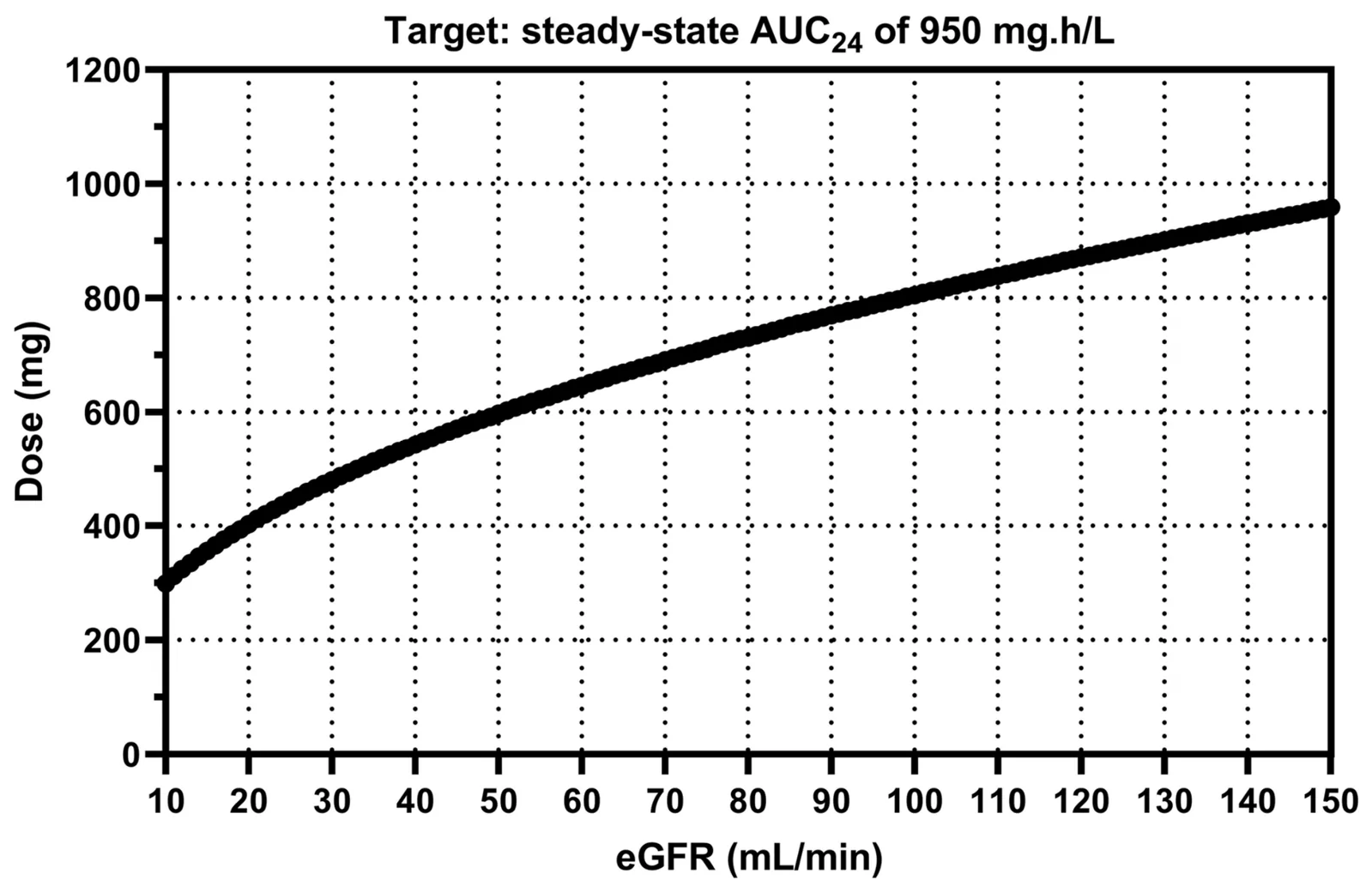
Nomogram for the calculation of fluconazole maintenance dose, administered once daily via 1 h intravenous infusion, based on patient’s estimated glomerular filtration rate (eGFR).

To facilitate its clinical application, this equation was translated into a practical dosing nomogram specifying the optimal daily fluconazole dose as a function of eGFR (Figure 3). The calculated daily dose should subsequently be divided according to the intended dosing interval and adjusted, if necessary, to the available intravenous dosage strengths. Since the simulation indicated a relatively prolonged time to reach steady-state, the administration of a loading dose equivalent to the 2.8× maintenance dose was simulated. The loading dose multiplier was derived from the ratio between the AUC_24_ [median (90%CI) of 887.66 (843.22–939.16)] on day 10 of therapy (steady state) and the AUC_24_ [median (90%CI) of 318.09 (292.11–343.43)] on day 1 of therapy.

Table 3 provides an overview of the PTA values for the efficacy PK/PD target (using MIC values of 2 and 4 mg/L) and for the toxicity threshold across the evaluated fluconazole dosing regimens.

## 4. Discussion

We developed a population pharmacokinetic model of intravenous fluconazole based on 116 hospitalized patients, including both ICU and non-ICU populations, who had TDM samples available. The model allowed us to evaluate multiple dosing regimens and to propose a simple eGFR-based dosing strategy that accounts for the wide spectrum of renal functions observed in this cohort.

A one-compartment model was selected because the retrospective TDM dataset contained predominantly one serum fluconazole concentration per dosing interval, precluding reliable estimation of intercompartmental distribution parameters. Under these conditions, a one-compartment model represented the most parsimonious structural model that could be robustly identified while adequately describing clinically relevant exposure for maintenance dose optimization. Although the sampling per individual was sparse, the distribution of samples across the dosing interval together with negligible η-shrinkage indicated that the available data were sufficiently informative for estimation of the population pharmacokinetic parameters and their inter-individual variability. Potential covariates were systematically evaluated, and eGFR emerged as the principal determinant of CL, while ABW was the most appropriate covariate for Vd. This is mechanistically consistent with the known pharmacology of fluconazole, which is predominantly renally eliminated in unchanged form and distributes extensively into total body water [7].

Our findings are broadly consistent with previously published population pharmacokinetic models. Matusik et al. demonstrated that creatinine clearance is a major determinant of fluconazole clearance, highlighting renal function rather than ICU status as the primary driver of interindividual variability [12]. Similarly, Van Daele et al. identified augmented renal clearance (ARC) and continuous veno-venous hemofiltration as factors that reduce fluconazole trough concentrations and exposure in critically ill patients [16]. Patel et al. developed a two-compartment model in critically ill adults using intensive pharmacokinetic sampling and identified creatinine clearance as the principal predictor of clearance [20], whereas Watt et al. reported a two-compartment model in critically ill pediatric patients with comparable findings regarding the importance of renal function [21]. The difference in structural model complexity most likely reflects differences in study design rather than underlying pharmacology. Both Patel et al. and Watt et al. employed rich pharmacokinetic sampling that enabled characterization of the distribution phase, whereas our model was developed from sparse routine TDM data, in which samples were obtained predominantly after distribution equilibrium had been reached.

Compared with these previous reports, the principal contribution of the present study lies not only in describing fluconazole pharmacokinetics but also in translating the population pharmacokinetic model into a clinically applicable dosing strategy. By combining model-based Monte Carlo simulations with an eGFR-guided dosing algorithm, we developed a simple dosing nomogram that maximizes the probability of PK/PD target attainment while limiting excessive exposure and can be readily implemented in routine hospital practice without specialized pharmacometric software.

We decided to use creatinine-based eGFR as a covariate for fluconazole CL because it allowed inclusion of the entire cohort. Among the 53 patients in whom both biomarkers were available, cystatin C-based eGFR values were systematically lower than creatinine-based estimates (median 46 vs. 90 mL/min), consistent with previous observations that these biomarkers may yield substantially different estimates of kidney function [22]. Nevertheless, both creatinine- and cystatin C-based eGFR showed comparable correlations with fluconazole clearance (Supplementary Figure S1). Because serum creatinine was repeatedly measured throughout therapy, whereas cystatin C was generally available only once, creatinine-based eGFR provided a more suitable time-varying covariate for population pharmacokinetic modeling. We acknowledge that creatinine-based estimates of GFR are influenced not only by glomerular filtration but also by tubular secretion of creatinine, which may vary between individuals because of physiological factors and genetic polymorphisms affecting renal transporters such as OCT2 [23,24]. However, serum creatinine is routinely measured in clinical practice and allows repeated estimation of renal function over time, making it the most practical covariate for model-informed dosing in our dataset. Monte Carlo simulations showed that eGFR-based dosing achieved the highest PTA for AUC_24_/MIC ≥ 100 at MICs of 2 and 4 mg/L (100% and 97.4%, respectively) without increasing the proportion of patients exceeding predefined toxicity thresholds. These findings support the hypothesis that fluconazole dosing can be individualized based on eGFR. Because fluconazole is predominantly renally excreted, adjusting doses according to a patient’s eGFR—not only in cases of renal impairment but also in patients with augmented renal clearance—may help achieve optimal PK/PD targets. Several studies have reported that guideline-based or simple weight-based dosing can result in underexposure in a substantial proportion of patients, particularly when treating less susceptible Candida strains [11,12,14]. Matusik et al. analyzed 52 patients (36 ICU, 16 non-ICU) using both parametric and non-parametric pharmacokinetic models. In their parametric model, only ICU status influenced CL (85% higher in ICU patients), whereas the non-parametric model identified creatinine clearance (Cockcroft–Gault with ideal body weight) as the key determinant [12]. This discrepancy highlights how model selection can affect covariate identification. Our results align with the non-parametric findings: renal function, rather than ICU status alone, is the primary driver of fluconazole CL variability. By using eGFR as a continuous covariate, our model enables precise dose individualization across the full spectrum of kidney function, avoiding crude categories such as “normal” versus “impaired,” and thus may prevent the common problem of underexposure. The practical implications of these findings are clear. In mixed ICU/non-ICU populations, fluconazole dosing can be tailored according to renal function. Standard fixed-dose or simple weight-based regimens, while convenient, often result in substantial underexposure. In contrast, our eGFR-based, model-informed dosing—implemented as a practical nomogram—facilitates early attainment of PK/PD targets. This approach is particularly valuable in empirical antifungal therapy, where the causative Candida species and its susceptibility are initially unknown; higher loading doses ensure adequate coverage against less-susceptible organisms (MIC up to 2–4 mg/L) while awaiting culture and susceptibility results. Our simulations indicate that the eGFR-optimized maintenance dose should be preceded by a 2.8-fold higher loading dose, which may raise concerns regarding toxicity. Because data on fluconazole toxicity thresholds remain limited, this strategy warrants further investigation. Anaissie et al. reported that high daily doses (>1600 mg) may be associated with toxic effects, while Matsumoto et al. observed a risk of neurotoxicity when trough fluconazole concentrations exceeded 80 mg/L [25,26]. On the other hand, recent studies suggest that higher loading doses are often necessary. Sandaradura et al. reported that effective loading doses range from 700 to 2400 mg, exceeding the commonly used 800 mg, and Novy et al. suggested an 18 mg/kg weight-based loading dose in critically ill patients receiving extracorporeal membrane oxygenation (ECMO) [11,13]. Notably, these studies indicate that administering a single initial loading dose is generally associated with a low likelihood of toxicity. Nevertheless, the loading dose strategy proposed in the present study is based solely on pharmacokinetic simulations and should therefore be regarded as hypothesis-generating. Prospective clinical studies are required to evaluate its safety and clinical effectiveness before implementation in routine practice. If future studies confirm the benefit of higher loading doses, strategies such as dose fractionation or prolonged infusion may be considered to reduce potential peak-related toxicity.

This study has several limitations related to its retrospective design. Fluconazole dosing and TDM sampling were clinically driven rather than protocolized; however, this variability is appropriately addressed by the population pharmacokinetic approach applied in the analysis. Due to sparse sampling during routine TDM procedures, patients receiving oral fluconazole or undergoing renal replacement therapy were excluded, as characterization of absorption variability and extracorporeal clearance would require more intensive sampling. Because of the limited sampling density, evaluation of a two-compartment model was also not feasible. Only total plasma fluconazole concentrations were measured, as assessment of the unbound fraction was not available. However, given the low protein binding of fluconazole, this limitation is unlikely to be clinically relevant, and total concentrations are also commonly used for PK/PD target evaluation [8]. Finally, the proposed eGFR-based dosing regimen is derived from simulations and requires prospective clinical validation to confirm its efficacy and safety. Nevertheless, the consistency of our findings with the previous literature supports the mechanistic plausibility and potential clinical utility of renal function-guided fluconazole dosing in hospitalized patients. Furthermore, patients with markedly augmented renal clearance were underrepresented in our cohort. Therefore, although the developed model may provide a rational framework for renal function-guided fluconazole dosing, its applicability in patients with very high eGFR values (e.g., >150 mL/min) remains uncertain and should be evaluated in dedicated cohorts enriched for patients with augmented renal clearance. Extrapolation of the current dosing nomogram beyond the observed range of renal function was not performed because it would exceed the data range used for model development.

## 5. Conclusions

We developed a population pharmacokinetic model of intravenous fluconazole that identified eGFR as the key covariate influencing fluconazole pharmacokinetics. Based on this model, an eGFR-derived dosing nomogram for initial therapy was proposed. Model-informed evaluations demonstrated that eGFR-based dosing achieved the highest probability of attaining the PK/PD target (AUC_24_/MIC ≥ 100) for MIC values of 2 and 4 mg/L compared with alternative dosing strategies, without increasing the proportion of patients exceeding predefined exposure-based toxicity threshold. These findings support renal function-guided fluconazole dosing as an effective and safe approach to reduce underexposure and improve the likelihood of therapeutic success in hospitalized patients.

## Figures and Tables

**Table 1:** Demographic, laboratory, and clinical data of the patients (*n* = 116).

Data	Unit	Value
Age	years	67 (58–74) [21–94]
Sex	/	78 males, 38 females
Height	cm	175 (169–180) [149–192]
Body weight	kg	85 (72–101) [43–178]
Adjusted body weight	kg	75 (68–84) [43–110]
Body surface area	m^2^	2.00 (1.84–2.20) [1.36–2.75]
Serum creatinine	µmol/L	70 (53–112) [29–483]
Creatinine-based eGFR	mL/min	86 (55–103) [8–160]
Serum creatinine ^1^	µmol/L	67 (55–118.5) [29–483]
Creatinine-based eGFR ^1^	mL/min	90 (51.5–109.5) [9–137]
Serum cystatin C ^1^	mg/L	1.41 (1.05–1.98) [0.66–5.20]
Cystatin C-based eGFR ^1^	mL/min	46 (31–71) [8–115]

Data are expressed as median (IQR) [range]. ^1^ Measured/calculated only in 53 patients in whom cystatin C was available.

**Table 2:** Estimates of the final fluconazole population pharmacokinetic model and the bootstrap results based on 500 simulations.

Parameter	Final Model		Bootstrap Analysis
	Estimate	R.S.E. (%)	Median (95% CI)
Fixed effects			
Vd = Vd_pop × (ABW/75) ^β_Vd_ABW^			
CL = CL_pop × (eGFR/90) ^β_CL_eGFR^			
Vd_pop (L)	41.58	6.98	42.12 (41.55–42.64)
β_Vd_ABW	2.28	16.0	2.24 (2.21–2.30)
CL_pop (L/h)	0.81	4.77	0.82 (0.82–0.83)
β_CL_eGFR	0.43	30.3	0.43 (0.41–0.45)
Between subject variability			
Vd	0.44	12.5	0.43 (0.42–0.44)
CL	0.25	19.0	0.29 (0.28–0.29)
β_CL_eGFR	1.24	19.8	1.22 (1.20–1.24)
Error model parameter			
Proportional	0.18	10.2	0.18 (0.18–0.18)

Vd—volume of distribution; CL—clearance; pop—typical value of parameter; β_CL_eGFR—effect of eGFR (mL/min) on CL; eGFR—glomerular filtration rate estimated according to CKD-EPI formula; β_Vd_ABW—effect of ABW (kg) on Vd; ABW—adjusted body weight; CI—confidence interval; R.S.E.—relative standard error.

**Table 3:** Probability of target attainment (%) for PK/PD target for efficacy (considering a MIC value of 2 or 4 mg/L) and for toxicity threshold at various fluconazole dosing regimens on the 10th day of fluconazole therapy (steady state).

Dosing Regimen	PK/PD Target for Efficacy of AUC_24_/MIC ≥ 100		Toxicity Threshold of AUC_24_ ˃ 1500 mg·h/L
	MIC = 2 mg/L	MIC = 4 mg/L	
LD 400 mg + MD 200 mg every 24 h	75.86 (69.83–81.90)%	13.79 (9.48–18.10)%	0.86 (0.00–2.59)%
LD 800 mg + MD 400 mg every 24 h	98.28 (95.69–100.00)%	75.86 (69.83–81.03)%	4.31 (1.72–6.90)%
400 mg every 12 h	99.14 (98.28–100.00)%	98.28 (95.69–100.00)%	14.66 (10.34–19.83)%
LD + MD nomogram	100.00 (98.28–100.00)%	97.41 (94.83–99.14)%	8.62 (5.17–13.36)%

Data are expressed as medians (90% CI) from replicates. AUC_24_—area under the concentration–time curve during a 24 h period; LD—loading dose; MD—maintenance dose; PK/PD—pharmacokinetic/pharmacodynamic.

## Data Availability

The data presented in this study are available on request from the corresponding author due to ethical reasons.

## Supplementary Materials

The following supporting information can be downloaded at https://www.mdpi.com/article/10.3390/pharmaceutics18080929/s1, Supplementary Figure S1: Preliminary graphical assessment and univariate association using Pearson’s correlation test of the effect of estimated glomerular filtration based on creatinine (eGFRcr) or cystatin C (eGFRcys) on individual fluconazole clearance values expressed as conditional mode. Supplementary Figure S2: Goodness-of-fit plots obtained from the final fluconazole population pharmacokinetic model: population and individual predictions of fluconazole versus observed concentrations. Supplementary Figure S3: Visual predictive check (shaded areas) for fluconazole serum concentration versus time for the final model.

## Abbreviations

The following abbreviations are used in this manuscript:

ABW	Adjusted body weight
AIC	Akaike information criteria
ARC	Augmented renal clearance
AUC_24_	24 h area under the curve
BIC	Bayesian information criteria
BSA	Body surface area
CI	Confidence interval
CL	Clearance
C_min_	Trough level
eGFR	Estimated glomerular filtration rate
GOF	Goodness of fit
ICU	Intensive care unit
IQR	Interquartile range
LD	Loading dose
MD	Maintenance dose
MIC	Minimal inhibition concentration
OFV	Objective function value
PK	Pharmacokinetic
PK/PD	Pharmacokinetic/pharmacodynamic
PTA	Probability of target attainment
R.S.E.	Relative standard error
Vd	Volume of distribution
VPC	Visual predictive check
