# Supplementary material for: Population Pharmacokinetic Modeling of Intravenous Fluconazole to Inform eGFR-Based Dosing: A Simulation Study Demonstrating Superiority over Standard Regimens
Source: Pharmaceutics. 2026 Jul 29;18(8):929. doi: 10.3390/pharmaceutics18080929 (PMC13516392; doi:10.3390/pharmaceutics18080929)

Population Pharmacokinetic Modeling of Intravenous Fluconazole to Inform eGFR-Based Dosing: A Simulation Study Demonstrating Superiority over Standard Regimens

Supplementary figure S1. Preliminary graphical assessment and univariate association using Pearson´s correlation test of the effect of estimated glomerular filtration based on creatinine (eGFRcr) or cystatin C (eGFRcys) on individual fluconazole clearance values expressed as conditional mode.

Regression lines are in red.

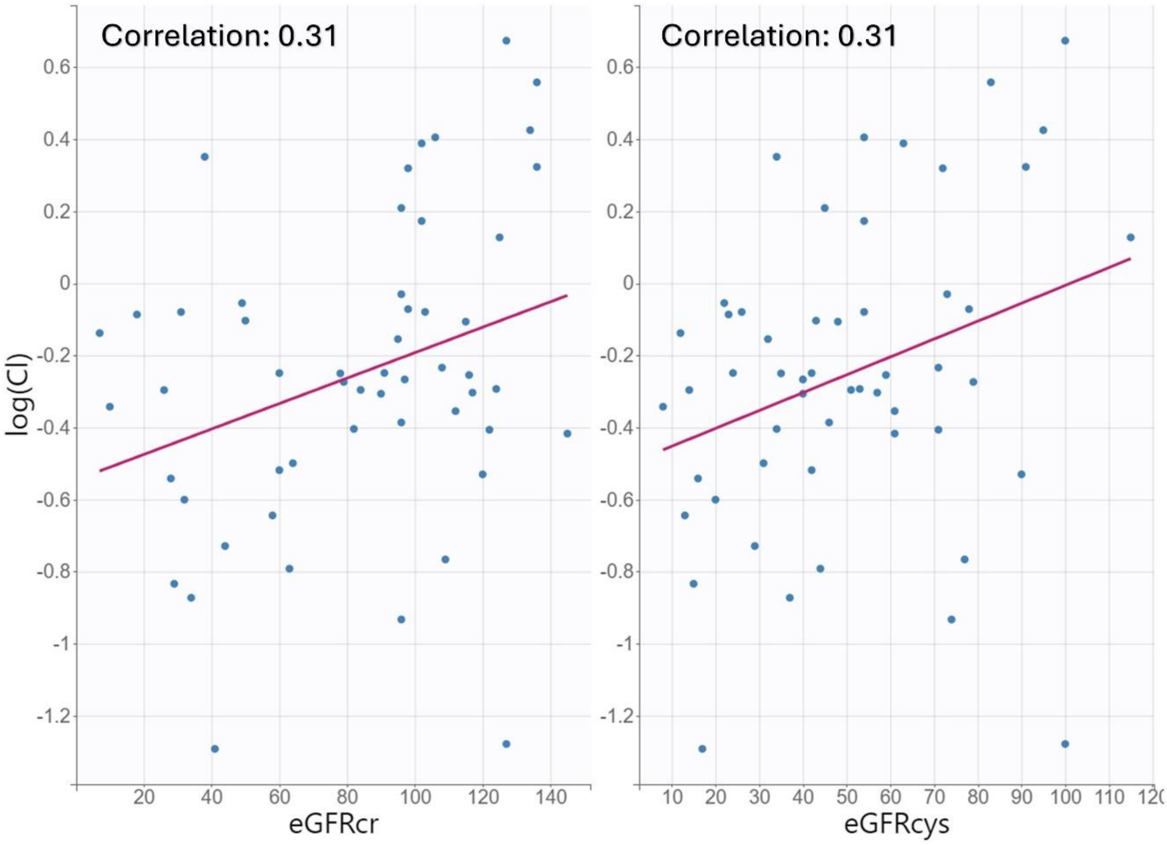

Supplementary figure S2. Goodness-of fit plots obtained from the final fluconazole population pharmacokinetic model: population and individual predictions of fluconazole versus observed concentrations.

Orange curves represent smoothing splines.

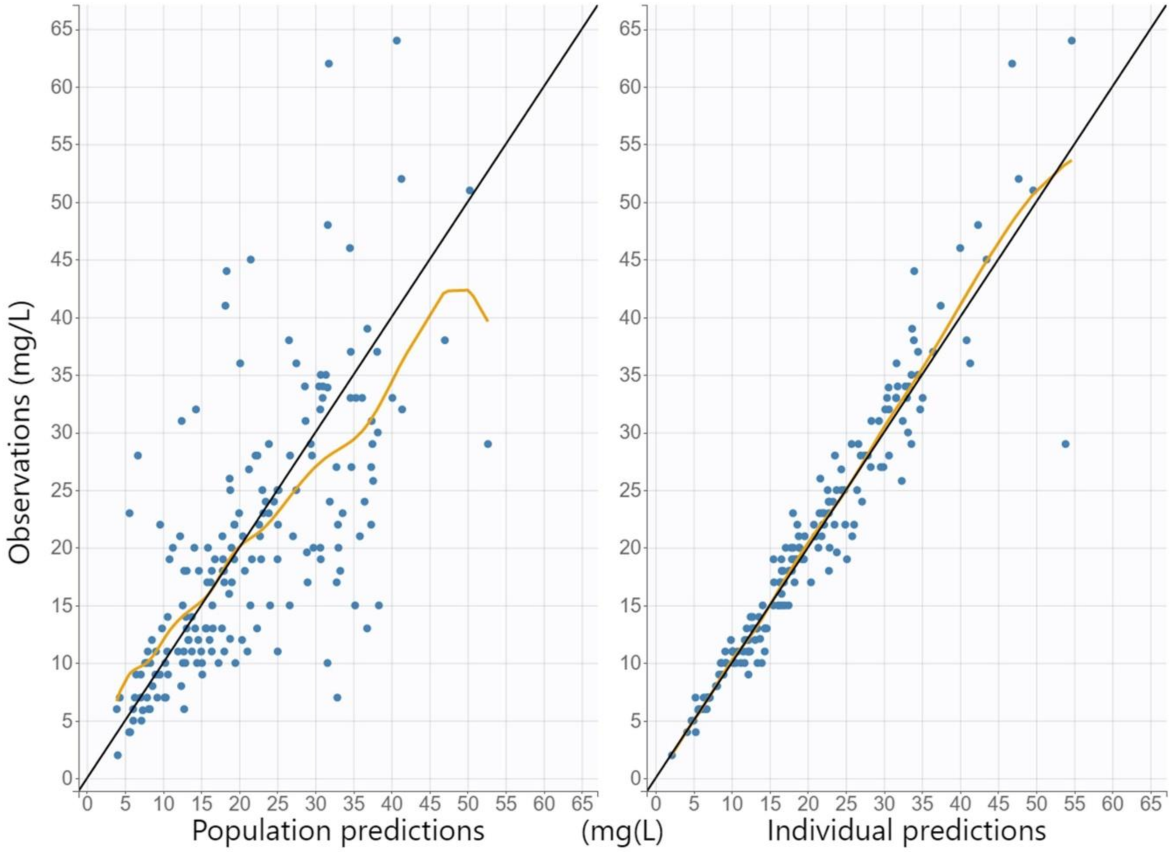

Supplementary figure S3. Visual predictive check (shaded areas) for fluconazole serum concentration versus time for the final model.

Solid blue lines represent the 10th, 50th, and 90th percentiles of the observed data. Shaded regions represent 90% confidence interval around the 10th (below blue region), 50th (pink region), and 90th (above blue region) percentiles of the simulated data.

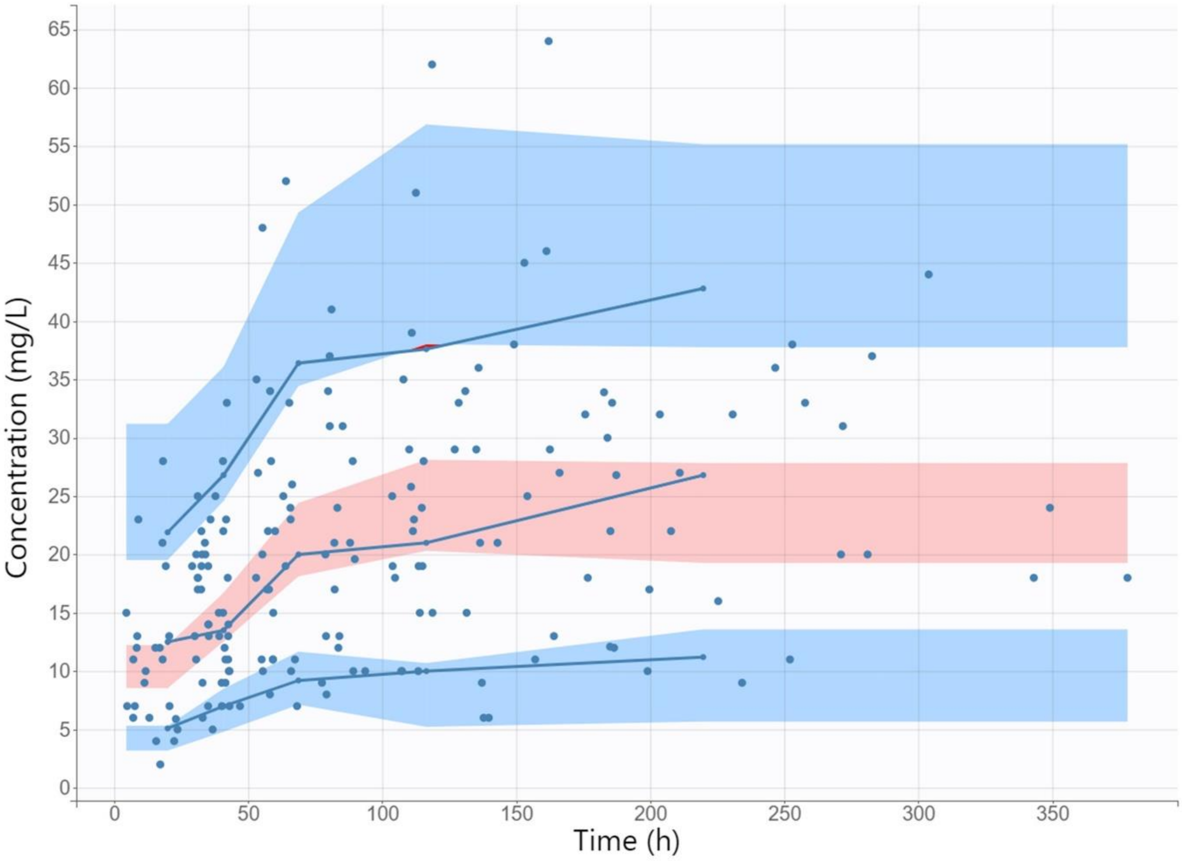

Supplement: Supplementary file 1 [file pharmaceutics-18-00929-s001.zip › pharmaceutics-4426843-supplementary.pdf]
